# Partitioning surface wave propagation on reconfigurable porous plane

**DOI:** 10.1038/s41598-023-50560-z

**Published:** 2024-01-02

**Authors:** Zhiyuan Chu, Kin-Fai Tong, Kai-Kit Wong, Chan-Byoung Chae, Yangyang Zhang

**Affiliations:** 1 Department of Electronic and Electrical Engineering, University College London, Torrington Place, London, WC1E 7JE UK; 2 School of Integrated Technology, Yonsei University, Seoul, 03722 Korea; 3 Kuang-Chi Science Limited, Hong Kong SAR, China

**Keywords:** Engineering, Materials science, Physics

## Abstract

This paper introduces a novel reconfigurable technique for partitioning the propagation of surface waves by utilizing a T-shaped structure and pathways established through the introduction of fluid metal or metal pins into evenly spaced cylindrical cavities within a porous surface wave platform. Notably, the co-printing of metal and dielectric materials via 3D printing is employed, resulting in an expedited fabrication process. Extensive 3D electromagnetic simulations and experimental investigations validate the proposed approach’s efficacy in achieving surface wave division while minimizing interference. The study encompasses an exploration of diverse power distribution ratios achievable within the distributed surface waves. Critical physical parameters of the T-junction are comprehensively examined, including partition depth, junction geometry, output port symmetry, and asymmetry. Additionally, the research delves into the frequency-dependent behaviours of asymmetric T-junctions and pathways. These findings establish the groundwork for adaptable architectures, facilitating concurrent communication among multiple devices within a unified surface wave communication network. This innovation holds potential to enhance various applications through improved communication capabilities.

## Introduction

Surface waves inherently propagate on two-dimensional (2D) dielectric-coated planar conductor plates, and offer lower decay when compared to traditional 3D wireless communications in the air. The classical result in^1^ has revealed that taking the advantage of its two-dimensional geometrical structure, unlike space waves with power attenuating according to the *square* of the propagation distance *d*, the power of surface waves drops only proportionally to *d* (not $d^2$). Additionally, as described by MacDonald functions^3^ and the clarifications provided in^2^, surface waves remain tightly bound to the propagation surface and are exponentially attenuated in directions away from the surface, this is a useful feature desirable for interference management in communications. It was recently suggested in^4^ that surface waves might play a major role in advancing reconfigurable intelligent surfaces (RISs) technology towards the sixth-generation (6G) mobile communications^5,6^.

Surface waves excited by finite rectangular apertures have an elliptical wavefront that originates from the transmitting terminal and spreads across the interface along the entire dielectric-clothed surface of propagation. Further signal strength improvement can be obtained by guiding the propagation direction of surface waves using some reconfigurable mechanisms. Various methods have been reported, such as printing periodic metasurface on a grounded dielectric slab^7^, holographic wideband beam scanning^8^, and leaky wave structures^9^. Techniques such as transformation cloak^10^ and the use of isotropic/anisotropic surfaces^11^ have also been implemented to reduce scattering losses of surface waves in three-dimensional space on curved and sharp surfaces. Besides, researchers have explored stacked Eaton lenses in the terahertz band^12^ and transformation optics^13^ to manipulate surface wave propagation directions through transmission in different dielectric media. Another approach involves applying fluid metals on reconfigurable surfaces to achieve flexible control of surface wave propagation^14^. Recent research has also extended the application of surface waves in wearable devices for body area networks^15,16^ and even on-chip network systems^17,18^.

However, these previous studies mainly focused on single-input single-output (SISO) architectures, and research addressing the challenges of enabling multiple simultaneous terminals on a shared surface wave communication platform is limited. Traditional wired communications uses fixed power dividers to create new interfaces^19^, but such inflexibility hampers the adaptability of a smart communication system. To fill this gap, this paper aims to demonstrate the feasibility of dynamically distributing surface wave into multiple pathways using a reconfigurable T-junction on a shared platform. The proposed approach involves pumping conductive fluid metal or inserting metal pins, into the small evenly distributed cylindrical cavities on the surface to create the splitting pins in the T-junctions at desired positions. We explore the arrangements of splitting pins through full electromagnetic (EM) simulations and experiments. Different positions of these pins in the T-junctions are investigated to evaluate the power division of surface waves and insertion loss at the junctions. Furthermore, we examine the power division ratio and the frequency selectivity of surface waves based on asymmetric splitting pin arrangements and choice of the pathway width.

This paper has made two major contributions. First of all, we investigate the effect of different T-junction parameters, including, (1) splitting depth: the extending distance of splitting pins from the bottom wall of a T-junction; (2) junction shape: the distributions of splitting pins in the junction; (3) symmetry: the symmetric splitting pins geometry; and (4) asymmetry: the asymmetric splitting pins geometry with different exit pathway widths. Secondly, we demonstrate the variable power division and frequency selectivity of surface wave pathways by creating asymmetric T-junction geometries. These results confirm the feasibility of surface wave applications in which multiple devices simultaneously access a shared surface wave communication network and require flexible control of the power and frequency of signal over different pathways.

## Reconfigurable porous surface

### Basic geometry of guided pathways

**Figure 1:**
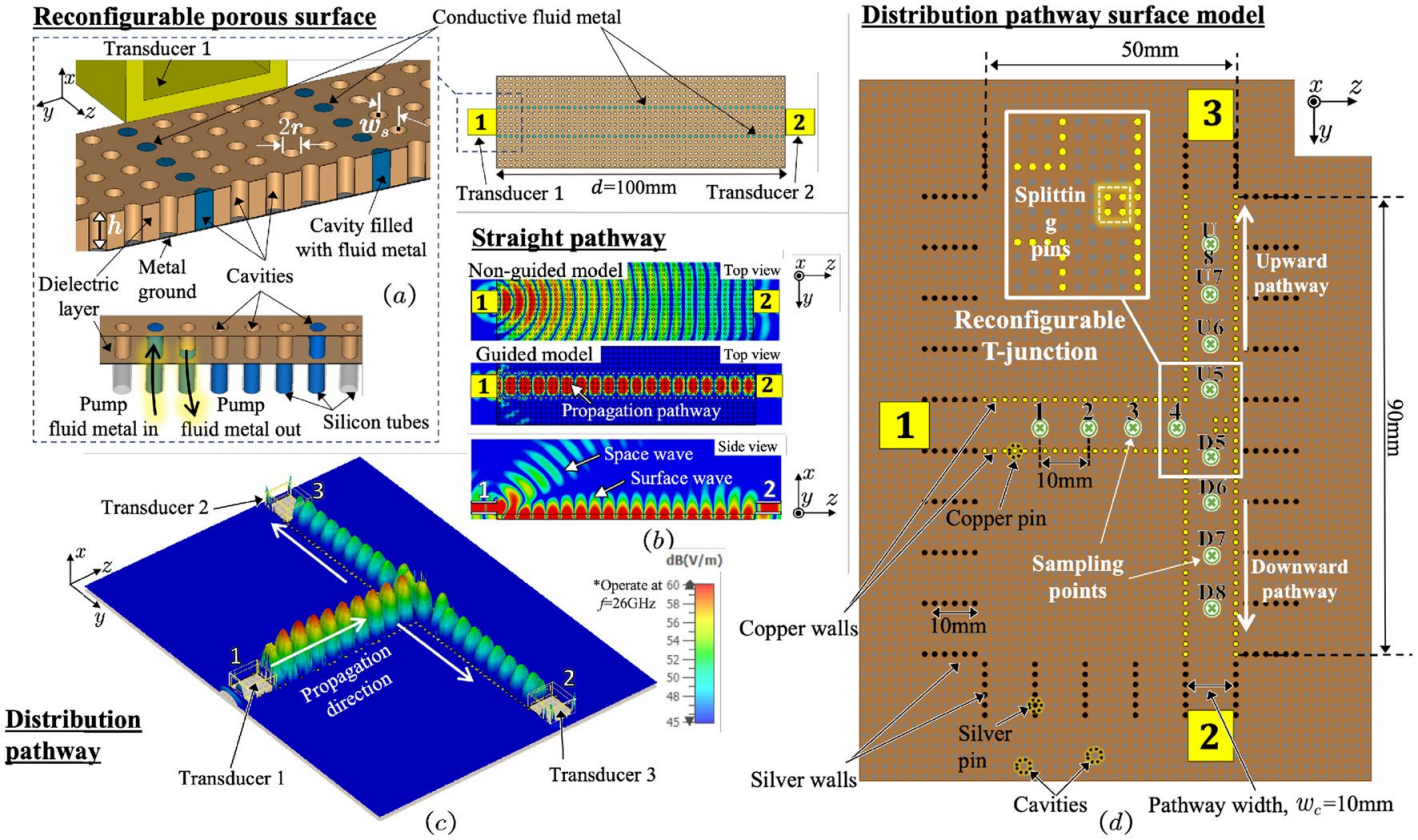
(**a**) Illustration of the proposed reconfigurable porous surface with conductive fluid metal filled at selected cavities and its working mechanism of dynamically creating propagation pathways. The corresponding simulation results for (**b**) the straight pathway in non-guided and guided models with a propagation distance *d* of $100\,\textrm{mm}$ and (**c**) the distribution pathway at $26\,\textrm{GHz}$ as well as (**d**) its surface model where the sampling points, 1–4, $\textrm{U}5$–$\textrm{U}8$ and $\textrm{D}5$–$\textrm{D}8$, of a $10\,\textrm{mm}$ interval are added along the distribution pathway with a reconfigurable T-junction.

Figure 1a shows the simulation model of reconfigurable surface wave platform geometry which consists of a top porous dielectric layer and a conductive metal ground as proposed in^14^. In the case of dynamic pathway creations and withdrawals, conductive fluid metal can be introduced from the back of the metal ground though silicon tubes into the evenly distributed cylindrical cavities to create the ‘pins’ which are connected to the metal ground as shown in Fig. 1d. Metal pins, such as copper or 3D-printed silver pins, can also be used and are used in the measurements in this paper to demonstrate the reconfigurability concept. A column of pins assemble a metal ’pin wall’ (Throughout this paper, we use the term ‘pin wall’ to mean a series of lined-up cavities filled with fluid metal/metal pins within the dielectric layer.) inside the dielectric layer. Then the reconfigurable propagation pathway can be created by a pair of pin walls for guiding the surface wave, whilst the reconfigurable T-shaped surface wave dividers are created by introducing metal pins into the vacant cavities in desirable positions at the T-junction in a similar way. The time domain solver of the commercial 3D electromagnetic simulation and analysis software CST Studio Suite (version 2022) is used in the modelling.

The surface wave propagation in non-guided and guided geometries are shown in Fig. 1b. The surface wave excited by Transducer 1 propagates towards Transducer 2, i.e., in the $+z$ direction along the dielectric surface in an open environment. From the top view of the non-guided model where all the cavities in the surface are vacant, it can be observed that the surface wave spreads across the entire surface. By contrast, a straight propagation pathway is created by two metal pin walls as described in the guided model, in which the surface wave is confined between the pin walls and propagates along the straight pathway with very limited leakage outside the pathway, indicating that this reconfigurable surface can create a highly efficient and isolated flexible surface wave pathway. Moreover, from the side view, we can observe that most of the wave excited from Transducer 1 is converted into surface wave and tightly bound to the surface, while only a small portion of the wave diverges into the air. The results in^20^ have demonstrated that bespoke surface wave transducers could maximize the excitation efficiency and suppress the space wave radiated from the transducer, therefore we focus more on discussing the reconfigurable divider concept in this paper.

### Surface impedance and approximated relative permittivity

The surface impedance $Z_s$ at the air-dielectric interface is defined as^21^

1 $$\begin{aligned} Z_s=\omega \mu _0\frac{\Delta }{2}+j\omega \mu _0\left[ \frac{\left( \varepsilon _r-1 \right) }{\varepsilon _r}h+\frac{\Delta }{2} \right] , \end{aligned}$$

where *h* and $\varepsilon _r$ represent the thickness and relative permittivity of the dielectric layer, respectively, $\mu _0=4\pi \times 10^{-7}\,\mathrm{H/m}$ is the vacuum permeability and

2 $$\begin{aligned} \Delta =\sqrt{\frac{2}{\omega \mu _0\sigma _m}}, \end{aligned}$$

in which $\Delta$ and $\sigma _m$ are the skin depth and electrical conductivity of the conductive metal ground, respectively. When the reactive component of surface impedance reaches an appropriate value, around $j250 \Omega$, a maximum amount of surface waves will be closely confined to the air-dielectric interface^20^. In the reconfigurable porous surface with even cavity distribution, the density of cavity affects the relative permittivity of the dielectric layer $\varepsilon _r$ and it can be approximated to be an effective value $\varepsilon _r^{{\rm eff}}$ as^22^

3 $$\begin{aligned} \varepsilon _r^{{\rm eff}}=\frac{\varepsilon _r\left[ 1+3\varepsilon _r+3\rho (1-\varepsilon _r)\right] }{1+3\varepsilon _r +\rho (\varepsilon _r-1)}, \end{aligned}$$

where

4 $$\begin{aligned} \rho =\frac{{S_{\rm cavity}}}{{S_{\rm surface}}} \end{aligned}$$

is the porosity, with $S_{{\rm cavity}}$ being the circular surface area of the cavity and $S_{{\rm surface}}$ being the total top surface area of the dielectric layer.

### Reconfigurable pathways

Conductive fluid metals, such as Galinstan, exhibit low adhesiveness and high fluidity, making them widely used in micro-fluidics and fluid antennas^23^. As shown in Fig. 1a and d, in this reconfigurable surface, conductive fluid metal is introduced into selected cavities through the silicon microtubes under the metal ground plate, forming two columns of metal pin walls with a pathway width $w_c$. The reconfigurability is realized by introducing liquid metal/metal pins in or out from the cavities to create or withdraw the dedicated pathways based on the communication requirements. Various reconfigurable pathways based on the specific arrangement of metal pins can divert the propagation directions, divide the surface wave into different power ratios or frequency bands. We will discuss these features in the subsequent sections.

### 3-dB power dividers

To illustrate the distribution characteristics under study, we have selected the simulation results of a T-shaped surface wave divider (depicted in Fig. 1c) and its corresponding geometric arrangement (shown in Fig. 1d) as an example. Within this illustrative scenario, Transducer 1 operates as the transmitter, while Transducers 2 and 3 are positioned symmetrically as receivers at the antipodal termini of the T-shaped pathway. After being excited and guided along the straight pathway in the $+z$-direction, the surface wave reaches the intersection point, i.e., the reconfigurable T-junction, where distinguished by strategically placed splitting pins. At this junction, the surface wave experiences a pivotal 90 $^{\circ }$ turn due to these pins, effectively dividing them into two separate branches. These branches continue along distinct routes, culminating in their arrival at Transducers 2 and 3 via pathways oriented in the $+y$ and $-y$-directions, respectively. Our focus in this section is to investigate how the arrangement of metal pins at the T-junction influences the power division between these split branches. Through the analysis of the $\textrm{S}_{j1}$ (where *j* = 2 or 3) across various pin distributions, the arrangement of the splitting pins (marked in Fig. 4) at the T-junction can result in different power divisions. We gain insights into optimizing the geometric setup for efficient surface wave distribution. Both in our simulations and measurements, we strategically position E-field sampling points (visualized as green dots in Fig. 1d) along the centerline of the surface wave pathway. These points, spaced at 10-mm intervals, span the region from 1-4U/D5-8 within the distributed pathways. Notably, Points 1 and U/D8 are situated over 20 mm away from the transducers to mitigate the impact of reflected waves at the transducers. In our experimental design, we opt for extended waveguides for the transducers, which helps minimize wave reflections originating from the adapters connected to the waveguide ends. It is important to highlight the significance of Points 4 and U/D5 at the T-junction. These points play a vital role in calculating and assessing the insertion losses, quantifying the attenuation experienced by the surface waves upon traversing the junction and subsequently splitting into the $+y$-(Transducer 2) and −y-directions (Transducer 3).

**Figure 2:**
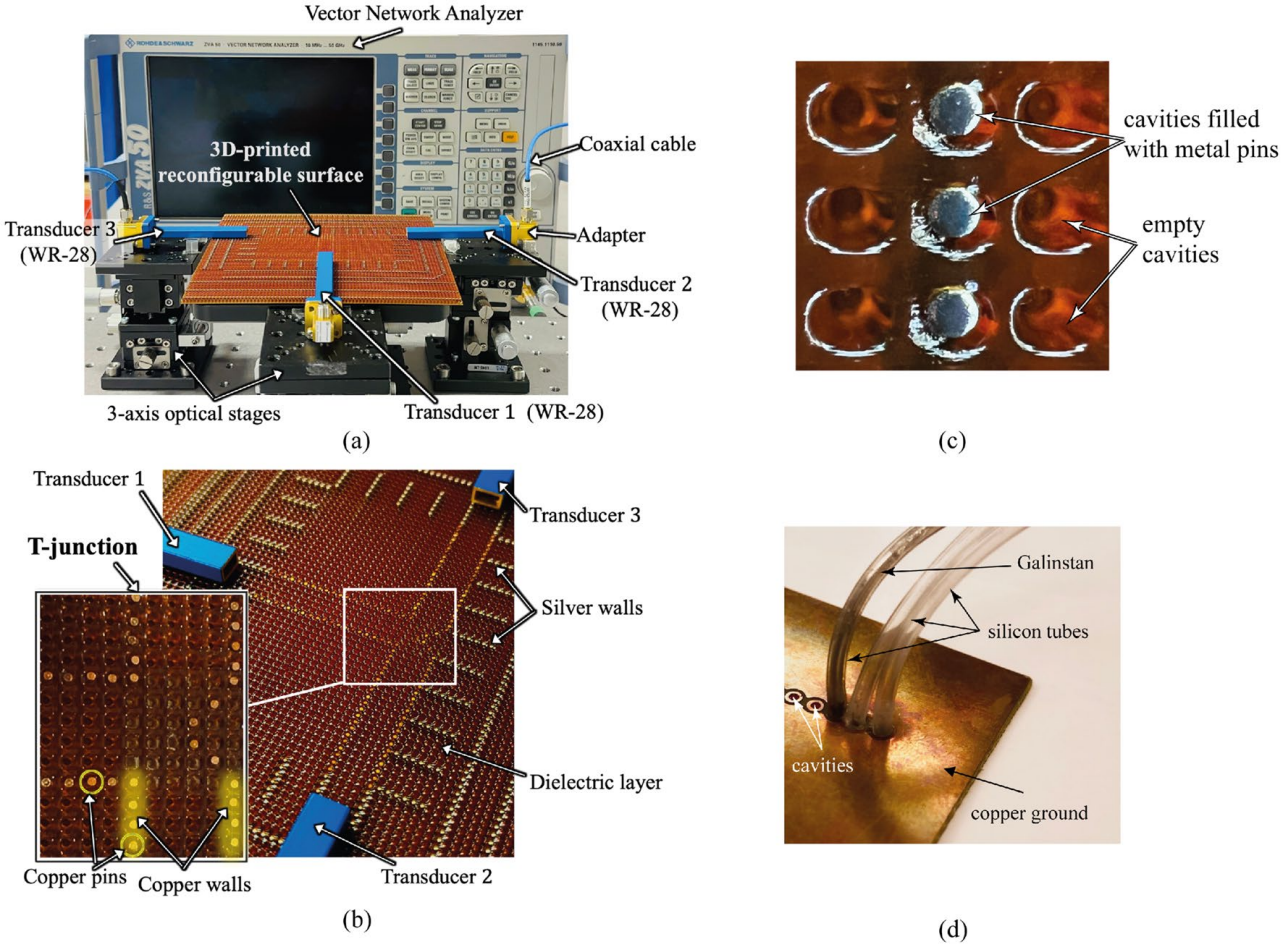
(**a**) The measurement setup of a 3D-printed reconfigurable surface prototype connected to a vector network analyzer, (**b**) the configuration of the T-junction, (**c**) zoomed photo of 3D-printed prototype, (**d**) example of filling mechanism of the fluid metal in vacant cavities.

**Figure 3:**
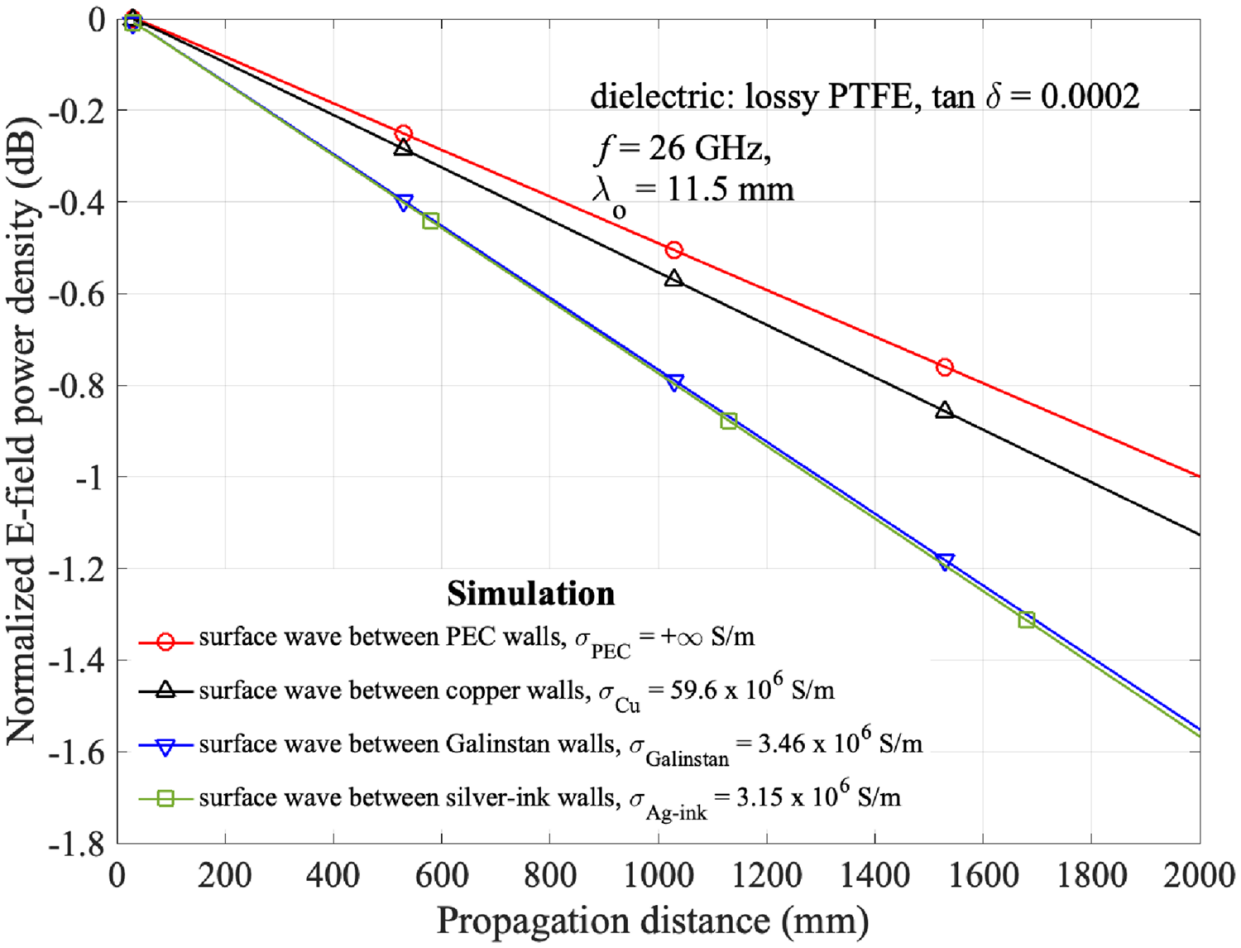
Decay of E-field power density of different metal walls in a straight pathway on a lossy PTFE surface.

**Figure 4:**
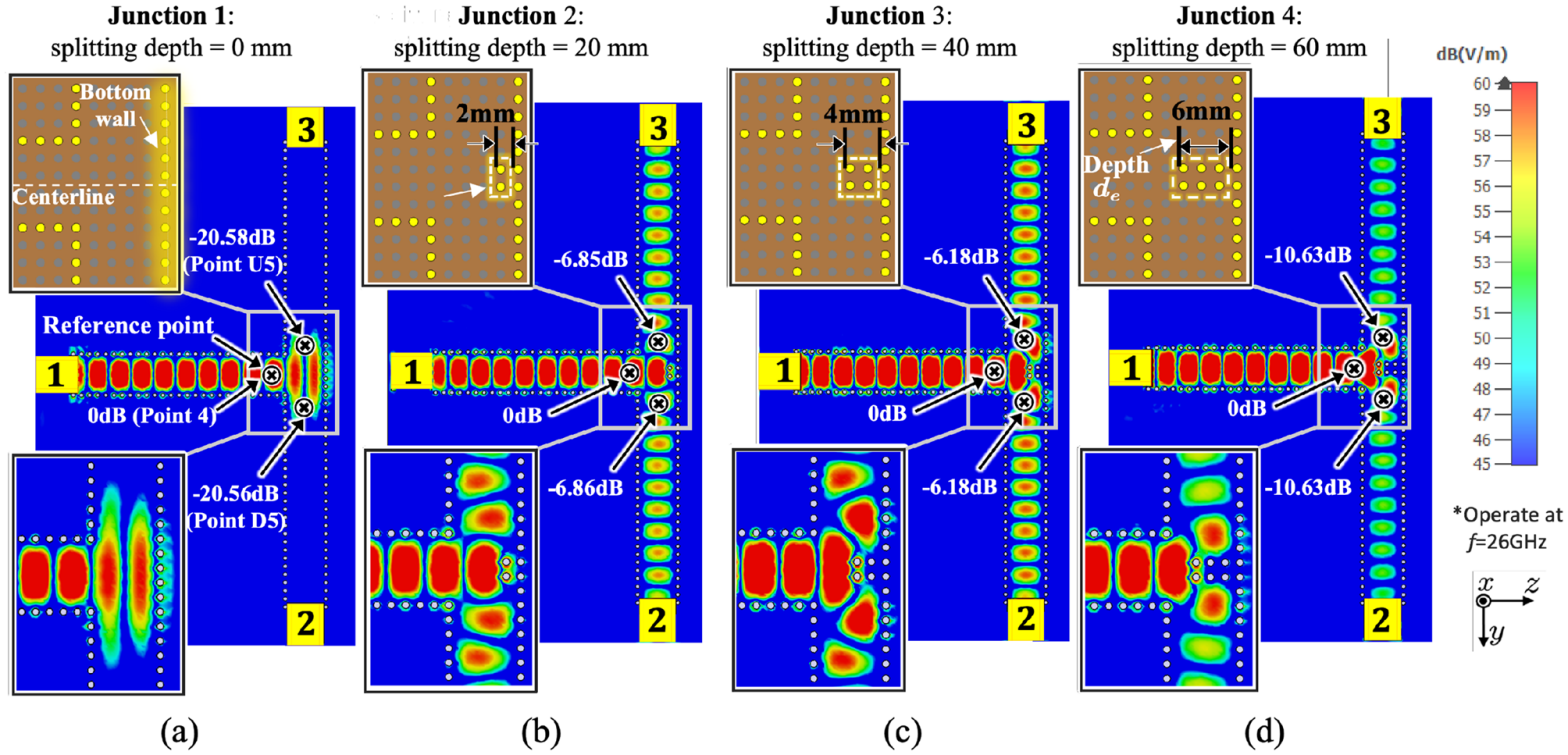
Illustration of the distribution pathway performances of a symmetric geometry in (**a**) Junction 1 without splitting pins, i.e., depth $d_e = 0$ mm, and Junctions 2–4 configured with the splitting pins with $d_e$ of (**b**) 2 mm, (**c**) 4 mm, and (**d**) 6 mm at 26 GHz.

## Results and discussion

### Measurement setup

Figure 2a shows the measurement setup of the reconfigurable surface wave platform prototype connected to a vector network analyzer (VNA) via the transducers (WR-28 waveguide), waveguide-to-coaxial adaptors, and coaxial cables for measuring the S-parameters. The VNA is first calibrated with open-circuit, short-circuit and 50 $\mathrm{\Omega }$ load standards in the interested frequency ranges at room temperature, i.e., 20 $^\circ \textrm{C}$. As the VNA only equips with two ports, either Transducer 2 or 3 is terminated with a 50$\mathrm{\Omega }$ load when the other port is being measured. The 3D-printer used in this project^24^ prints silver ink ($\sigma _s=3.15\times 10^6\,\mathrm{S/m}$) and dielectric resin ($\varepsilon _r=2.796$, $\tan \delta =0.0155$ at $26\,\textrm{GHz}$) simultaneously. As shown in Fig. 2b silver ink pins are printed into the cylindrical cavities inside the dielectric layer and connected to a thin silver ground plane on the bottom side for saving the fabrication time of surface wave platform. Three WR-28 rectangular waveguides with a wall thickness of $1\, \textrm{mm}$ are selected as the transducers. The transducers are mounted on the 3-axis optical linear stages for precise motion to different positions. Copper pins with the same radius as the circular cavity are used in the middle customized surface to demonstrate the reconfigurability of the surface. Although the electrical conductivity of the copper pins used in the experiment is different from common liquid metals such as Galinstan and the sliver ink used in the 3D-printed geometry, it can be observed in Fig. 3 that the difference in the rate of E-field power density decay between galinstan/silver ink and copper is about 0.2 dB/m or 0.0023 dB/$\lambda _o$ at 26 GHz. We consider this difference is acceptable for the purpose of demonstrating the reconfigurability of pathways in this paper. it has also been verified that the conductivity of pins does not significantly affect the measured results^25^. The electrical conductivity of different metal pins and other specific parameter values are listed in Table 1. The experiments are performed in room temperature and relative humidity, they are within the operating temperature and humidity of 3D-print dielectric and silver ink, i.e. 18–22 $^\circ$C and 35–55% non-condensing. To minimize the experimental errors in the measurement, the VNA is calibrated in the beginning of each measurement and five sets of data are measured and averaged in the experiments. In addition, the RF cables are fixed on the optical table by using sticky taps to minimize any movement during the measurement and the positions of the transducers are precisely controlled by the 3-axis optical linear stages. From the magnified photo shown in Fig. 2c, the top surface of the 3D-print platform is not perfectly flat, it expects that the surface roughness will introduce extra loss . However, when compared to the simulation results, we consider that such loss is acceptable.

**Table 1:** Key parameters in the measurements and simulations.

Surface parameters	Value
Radius of cavity/metal pin, *r*	0.5 mm
Center-to-center separation between cavities, $w_s$	2 mm
Electrical conductivity of silver ink, $\sigma _s$	3.15 $\times 10^6$ S/m
Electrical conductivity of Galinstan, $\sigma _g$	3.46 $\times 10^6$ S/m
Electrical conductivity of copper pin, $\sigma _c$	59.6 $\times 10^6$ S/m
Thickness of the metal ground, $h_m$	0.05 mm
Relative permittivity of the dielectric layer, $\varepsilon _r$	2.8
Effective permittivity of the dielectric layer, $\varepsilon _r^{{\rm eff}}$	2.4
Dielectric loss tangent, $\tan \delta$	0.0155 at 26 GHz
Thickness of the dielectric layer, *h*	2 mm
Surface impedance, $Z_s$	$j240\,\mathrm{\Omega }$ at 26 GHz
Pathway width, $w_c$	10 mm
Depth of splitting pins, $d_e$	0, 2, 4, 6 mm
Vacuum permittivity, $\varepsilon _0$	8.854$\times 10^{-12}$ F/m
Vacuum permeability, $\mu _0$	4$\pi \times 10^{-7}$ H/m

**Transducer parameters**	Value
Targeted operating frequency, *f*	26 GHz
Transducer (WR-28) frequency band, $f_b$	21–42 GHz
Height of transducer aperture, $h_a$	3.566 mm
Width of transducer aperture, $w_a$	7.112 mm
Thickness of transducer wall, $l_a$	1 mm
WR-28 to 2.92mm coaxial adaptor	26.5–40 GHz
	VSWR: 1.2:1 (Typ.)

### Symmetric divider

As depicted in Fig. 4, a symmetric (3-dB) divider is considered to explore the efficacy of T-junctions in distributing surface waves. Splitting pins are introduced at the T-junction and symmetrically distributed along the centerline. The parameter “Splitting Depth” refers to the distance of the splitting pins extending from the bottom wall of the junction towards the $-z$-direction. The splitting depth is increased from 0 mm at Junction 1–6 mm at Junction 4, with increments of 2 mm between each step. A reference point is labeled as Point 4, situated just before the surface wave enters the divider from Transducer 1. At this point, the normalized E-field power density is 0 dB. Additionally, we identify Point U5 and Point D5 as the locations where the surface wave exits the divider.

Based on the E-field distribution at Junction 1 shown in Fig. 4a, it can be observed that the surface waves are directly reflected at the bottom wall when the splitting pins are not introduced, resulting in a value of −20.58 dB at Points D/U8. This means that most of the surface waves are reflected back to Transducer 1, and very little surface waves can be guided to Transducers 2 and 3. In contrast, in Junctions 2 to 4, where splitting pins are introduced, the corresponding values at Points D/U8 are −6.85 dB, −6.18 dB, and −10.62 dB, respectively. The surface waves received by Transducers 2 and 3 are significantly increased when compared to that in Junction 1. This indicates that the splitting pins can realize surface wave division while reducing the reflection of surface waves at the junctions and increasing the signals at the receivers. It can also be seen that the E-field power at Points D8 and U8 in the junctions are consistent. It can be inferred that the symmetric geometry distributes surface waves equally along both the $+y$- and $-y$-directed pathways.

**Table 2:** The E-field power variations at T-junctions.

Model	*h* (mm)	Point 4$^\S$	Points D5/U5 (Ave.) (in dB)	Insertion Loss incurred (in dB)${\,}^\ddag$
Straight path	2.0	0	− 0.28	0.28
90$^{\circ }$-bend	1.5	0	− 5.55	5.55
90$^{\circ }$-bend	2.0	0	− 3.11	3.11
90$^{\circ }$-bend	2.5	0	− 3.63	3.63
Junction 1	2.0	0	− 20.56	17.75
Junction 2	2.0	0	− 6.86	3.75
Junction 3	2.0	0	− 6.18	3.07
Junction 4	2.0	0	− 10.62	7.51

${\,}^\S$The value at the reference Point 4 is normalized to 0 dB in each pathway.

${\,}^\ddag$The insertion loss (dB) incurred at the Junctions is determined by subtracting the E-field power at the 90$^\circ$-bend to that at Points D5/U5 located at exits of the reconfigurable T-junction.

**Figure 5:**
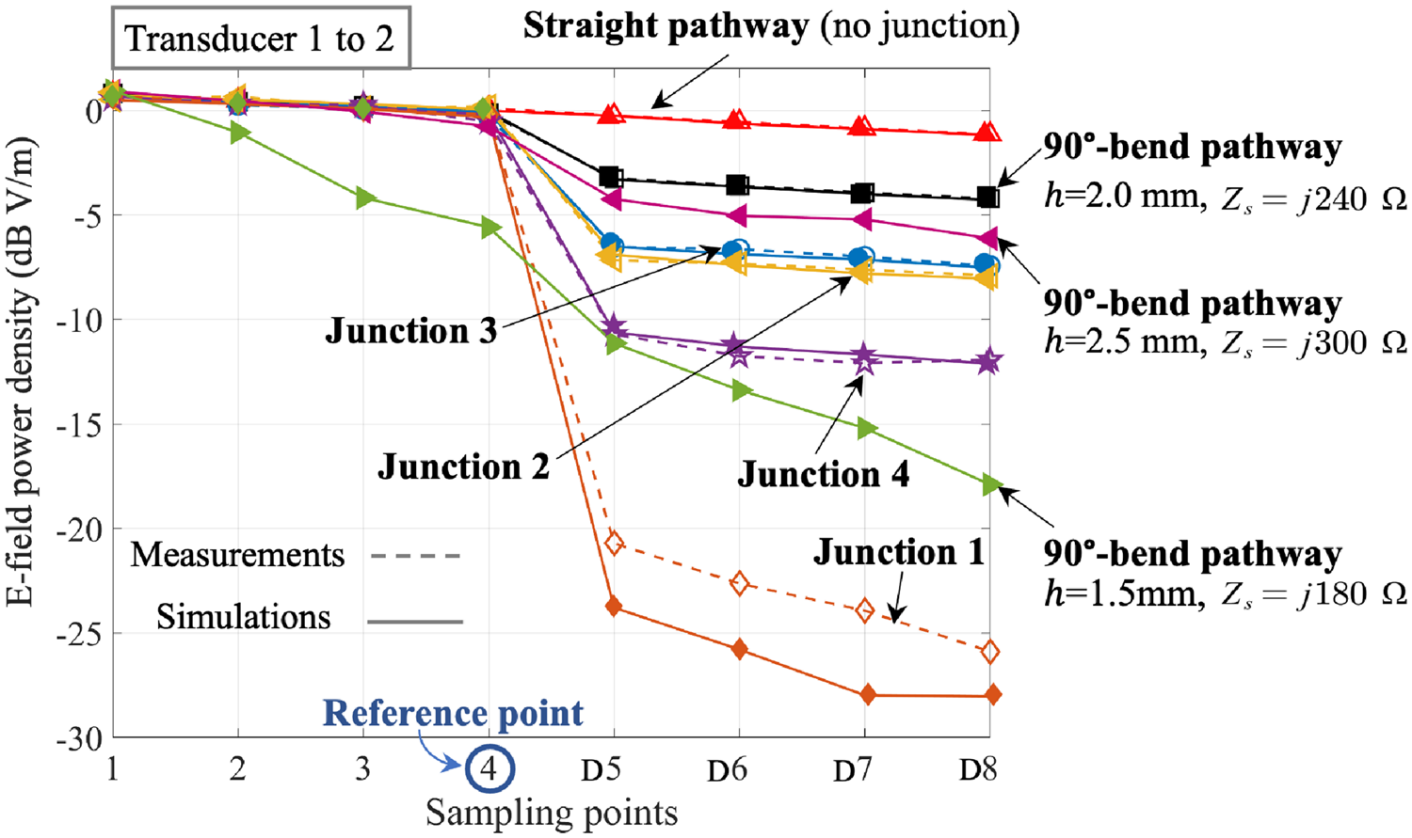
The comparison of the E-field power density (dB, V/m) attenuation at each sampling point in 1-$\textrm{D}8$ from Transducer 1 to 2 in measurement and simulation results at 26 GHz.

Figure 5 displays the normalized E-field power density (dB) at Points 1 to D8 along the centerline of distributed pathway from Transducers 1 to 2, at 26 GHz. Two reference models are used to study the performance of Junctions 1 to 4. The first model is a straight pathway without any junction, which has a decay of 0.322 dB/$$\lambda _o$$. Note that the 0.322dB/$$\lambda _o$$ decay is mainly caused by the dielectric loss tangent of the 3D-print resin, i.e., $$\tan \delta $$ = 0.0155 at 26 GHz, and it can be reduced by using low loss tangent materials such as conventional PTFE, i.e., $$\tan \delta $$ = 0.00022 at 26 GHz^26^. The second model on the other hand is a 90 $$^{\circ }$$-bend pathway with an insertion loss of 3.11 dB at the junction^14^. It serves as a reference for de-embedding the insertion loss incurred at 90$$^\circ $$-bend. By finding the E-field power differences between the 90$$^\circ $$-bend and the Junctions, we can calculate the power division achieved by the surface wave dividers. The attenuation between sampling points follows the same linear decay rate in the straight pathway. Additionally, the agreement between measurement and simulation results across the models confirms the accuracy of the simulations, except the higher measured $$\textrm{S}_{21}$$ at Junction 1. This discrepancy may be attributed to the reflection in the physical environment, causing the space wave to be received by Transducer 2, an effect not accounted for in the simulation where the boundary condition is set as open space without reflection. Same results are recorded from Points 1 to U8 on the pathway from Transducers 1 to 3. The impact of different dielectric thicknesses, i.e., *h* = 1.5 mm ( $$Z_s$$ = $$ j$$180 $$\Omega $$) and 2.5 mm ($$Z_s$$ = $$ j$$300 $$\Omega $$) has also been considered. As the corresponding surface impedance of the two thicknesses are deviated from the optimum value of $$Z_s$$ = $$ j$$250 $$\Omega $$^20^, higher decay rate along the pathway is observed along points 1 to 4, moreover, higher insertion losses at the 90$$^\circ $$-bends (at points U5 and D5) are resulted as shown in Fig. 5 and Table 2.

Table 2 also provides the measured E-field power at Points 4, D5, U5 at the T-junction and the insertion loss of each model. The symmetric geometry means that the values at D5 and U5 are nearly identical, and the insertion losses are obtained by subtracting the average E-field power at D5 and U5 from Point 4. After deducting the insertion loss of 3.11 dB at the 90$^\circ$-bend, a significant insertion loss of 17.75 dB is incurred in Junction 1, indicating that the model without the splitting pins cannot efficiently distribute the surface waves. The insertion losses are 6.86 dB in Junction 2 and 6.18 dB in Junction 3, respectively. In considering the 3.11 dB loss caused by the 90$^\circ$-bend, both Junctions 2 and 3 perform reasonably well as a 3-dB power divider, while Junction 4 may not be a good choice. Moreover, Junction 3 with the splitting pins of 4 mm depth exhibits the least insertion loss, suggesting that deeper splitting pins in Junction 3 perform slightly better than the smaller ones in Junction 2 by guiding the surface waves a slightly longer distance along the splitting pins at the T-junction, resulting in less reflection on the bottom wall. The relatively weaker distribution performance of Junction 4 can be attributed to the excessively long splitting pins, which further reduces the width of the exit at the T-junction, causing a certain amount of mis-match. Therefore, an appropriate splitting pin arrangement can achieve better surface wave distribution. Higher resolution, i.e., smaller than the existing 2 mm separation, of pin and cavity distributions will provide more freedom to further optimize the results by taking into account the surface geometry specific to the application scenarios.

### Flexible open and block pathways

**Figure 6:**
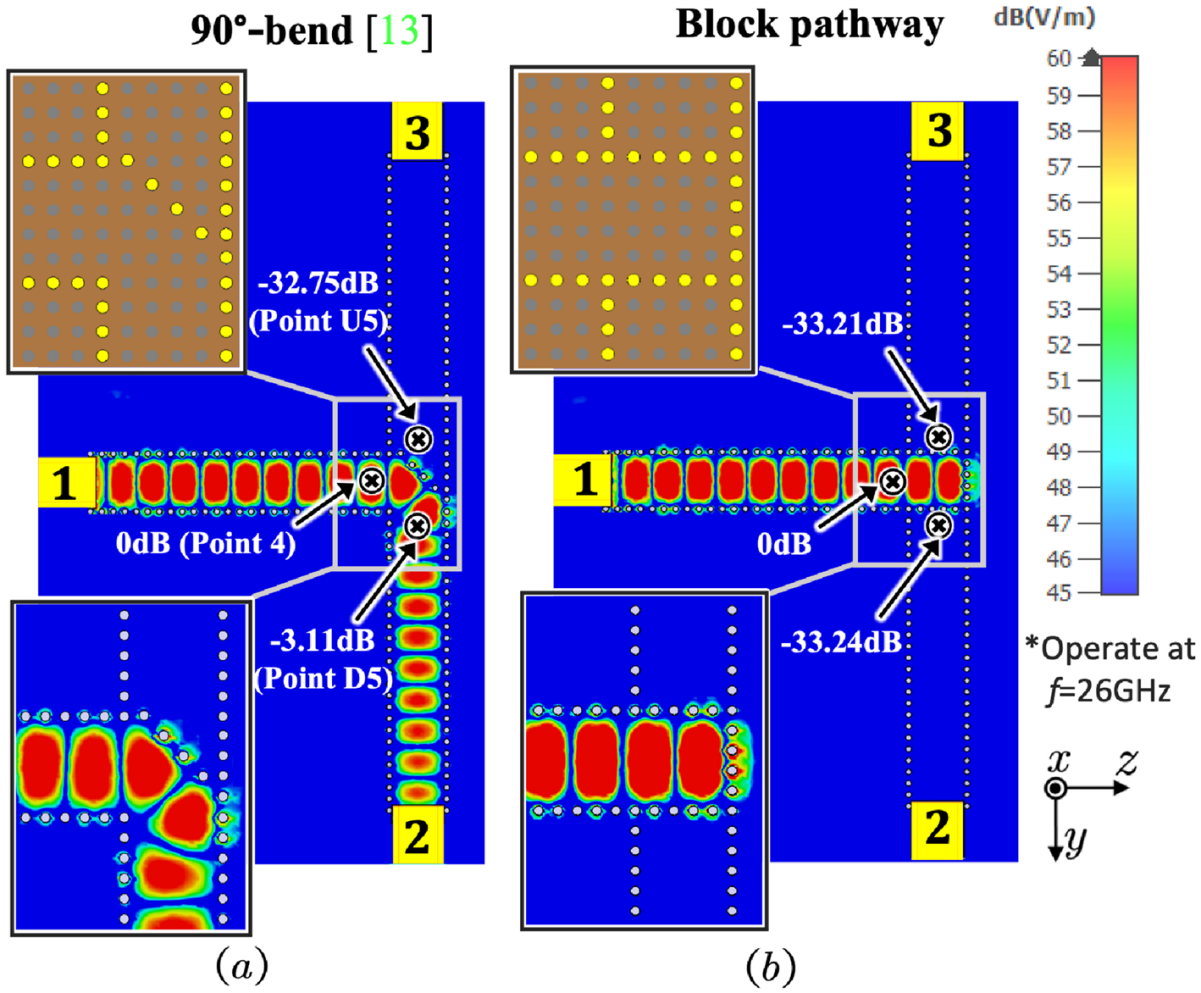
The E-field contour of (**a**) a 90$^\circ$-bend pathway and (**b**) a blocked pathway after the metal pins at the T-junction, operating at 26 GHz.

**Figure 7:**
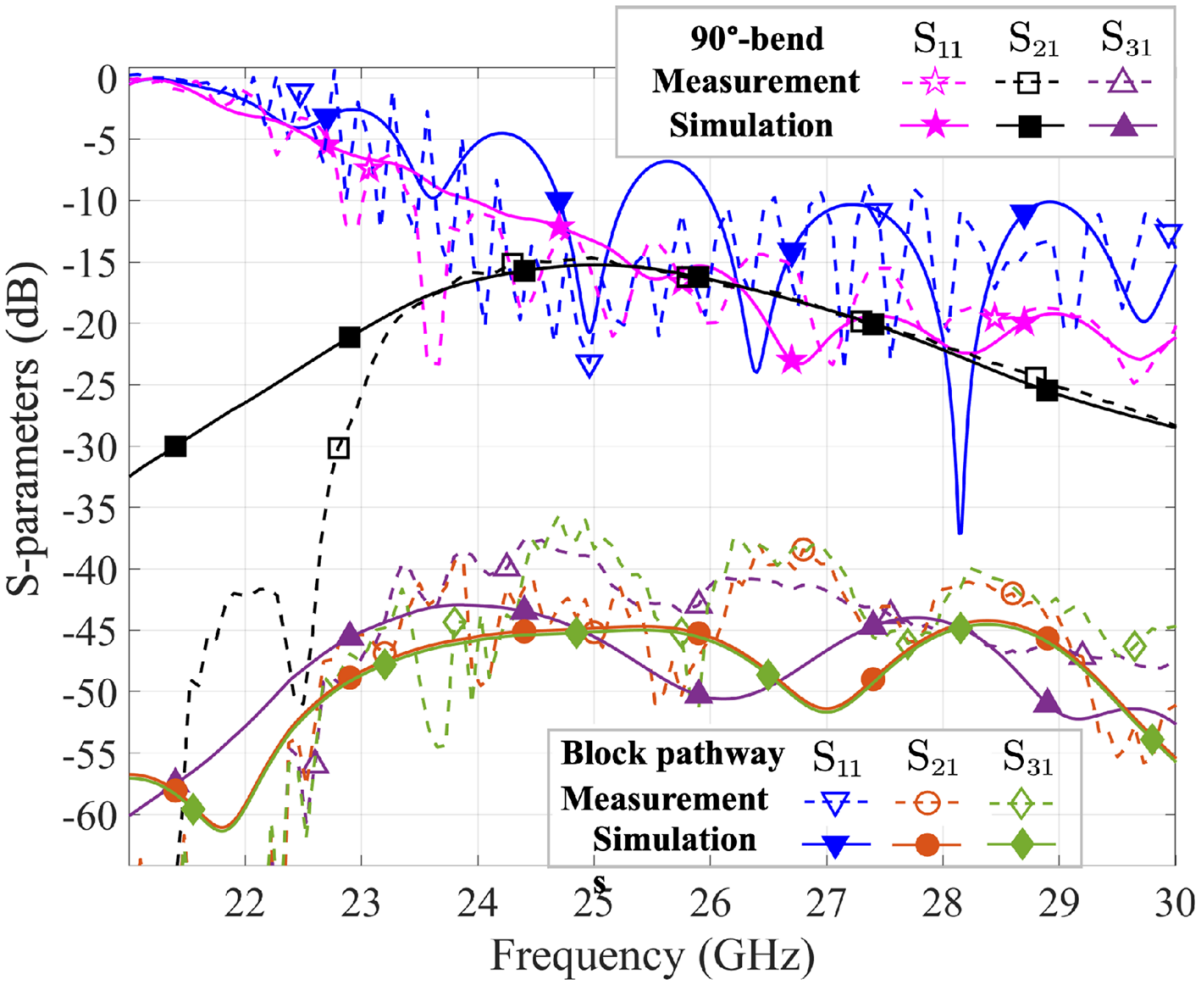
The $\textrm{S}_{11}$/$\textrm{S}_{21}$/$\textrm{S}_{31}$ ($\textrm{dB}$) results for a 90$^\circ$-bend pathway and a block pathway in the measurements and simulations.

Figure 6 provides the simulation results of a 90$^\circ$-bend pathway^14^ and a block pathway on the same surface at 26 GHz for reference. After introducing splitting pins to a 45$^\circ$ arrangement at the T-junction, the 90$^\circ$-bend pathway guides the surface waves only from Transducers 1 to 2 while blocks the wave to Transducer 3. The results indicate that there is a significant difference of nearly 29.6 dB between Transducers 2 and 3, with a value of $-\,3.114$ dB at the sampling point D5 and $-\,32.75$ dB at U5. In contrast, the block pathway blocks the propagation of the surface waves towards both transducers. The $\mathrm{S_{11}}$/$\mathrm{S_{21}}$/$\mathrm{S_{31}}$ curves in Fig. 7 illustrate that the $\mathrm{S_{31}}$ in the 90$^\circ$-bend pathway is about $-\,45$ dB, similar to the $\mathrm{S_{21}}$ and $\mathrm{S_{31}}$ in of the block pathway model, with a difference of nearly 30 dB from the $\mathrm{S_{21}}$ value in the 90$^\circ$-bend pathway. Also, the value of $\mathrm{S_{11}}$ in the block pathway is slightly higher than that in the 90$^\circ$-bend pathway, due to the presence of more reflections in the block pathway. Moreover, both the experimental and simulation results are in agreement. These results demonstrate that the surface wave pathway design can effectively guide and isolate surface waves, providing support for flexible control of surface wave propagation and mutual interference.

### Shape of T-junction

**Figure 8:**
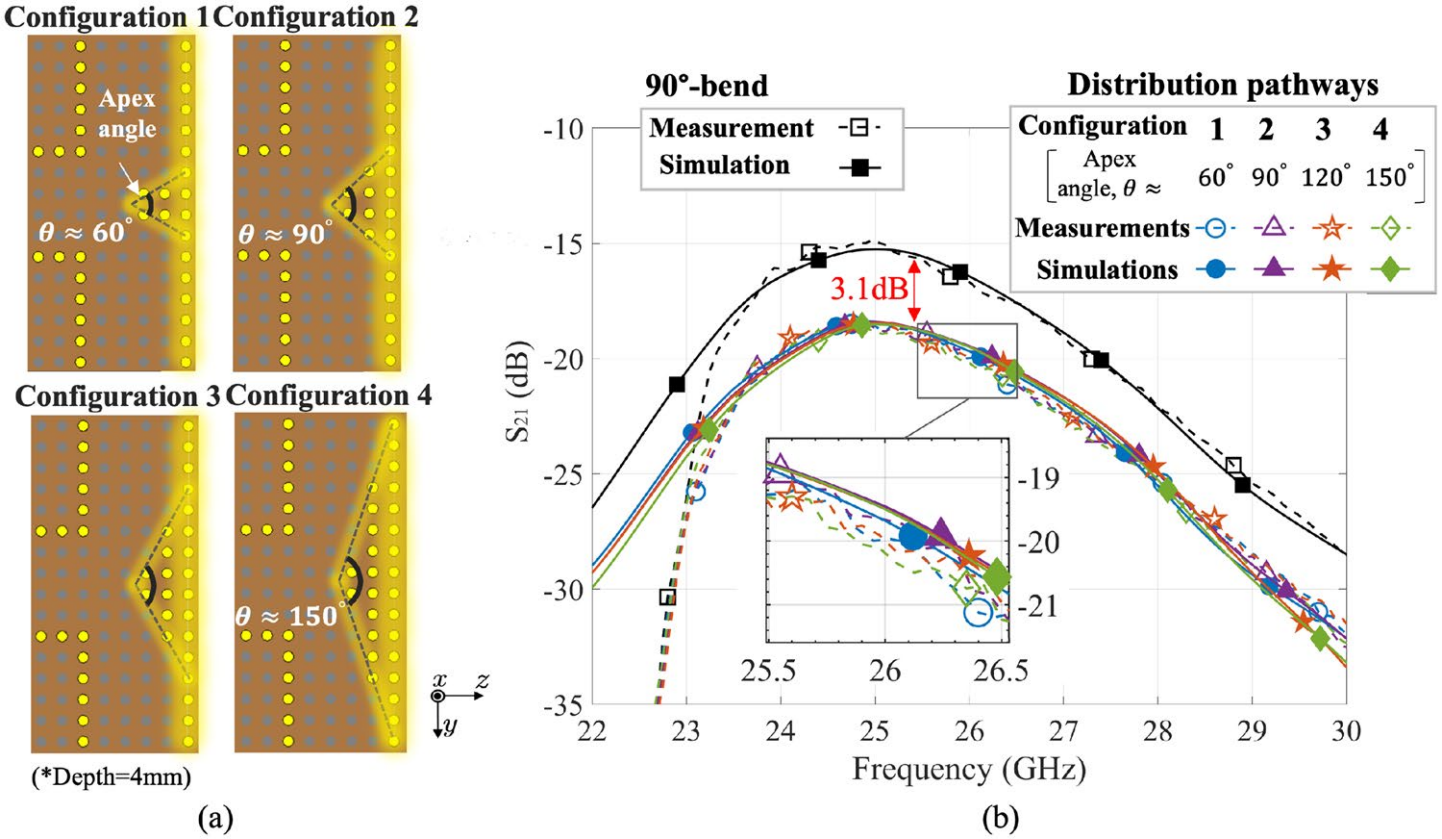
(**a**) The different symmetric configurations (Configurations 1–4) of splitting pins with an apex angle of around 60$^\circ$, 90$^\circ$, 120$^\circ$, and 150$^\circ$, respectively, and (**b**) their corresponding $\textrm{S}_{21}$ ($\textrm{dB}$) results in the measurements and simulations compared with those in the 90$^\circ$-bend pathway.

Further investigations are conducted to evaluate the effects of different shapes of the splitting pin arrangement in the T-junction. The reference model, Configuration 1, is characterized by a splitting depth of 4 mm, which gives the smallest insertion loss in “Symmetric divider” section, symmetrically located on the centerline. By gradually increasing the width of the base of the T-junction, Configurations 2–4 are obtained as depicted in Fig. 8a. The edges, created by joining the pins, together with the bottom wall, can be approximated as isosceles triangles with apex angles ($\theta$) of around 60$^\circ$, 90$^\circ$, 120$^\circ$, and 150$^\circ$, respectively. Figure 8b shows the measured and simulated $\textrm{S}_{21}$ across 22–30 GHz for different configurations, which have negligible differences of about 0.1 dB. The results show that changing the apex angles does not greatly affect the surface wave signal received at the Transducers 2 and 3, whereas the splitting depth of the T-junction has a much greater impact on the insertion loss. This means that we can simply use the basic Configuration 1 as a low insertion loss junction for distributing surface waves when the pins maintain an appropriate depth, such as 4 mm. Furthermore, the measured and simulated $\textrm{S}_{21}$ results agree well with each other in general, but the experimental $\textrm{S}_{21}$ values are lower than those in the simulations below approximately 23.5 GHz. This discrepancy may be attributed to the cutoff frequency of the coaxial-to-waveguide adapters used in the measurements^27^, which is not considered in the simulations where wideband waveguide ports are used to be directly connected to the transducers.

**Figure 9:**
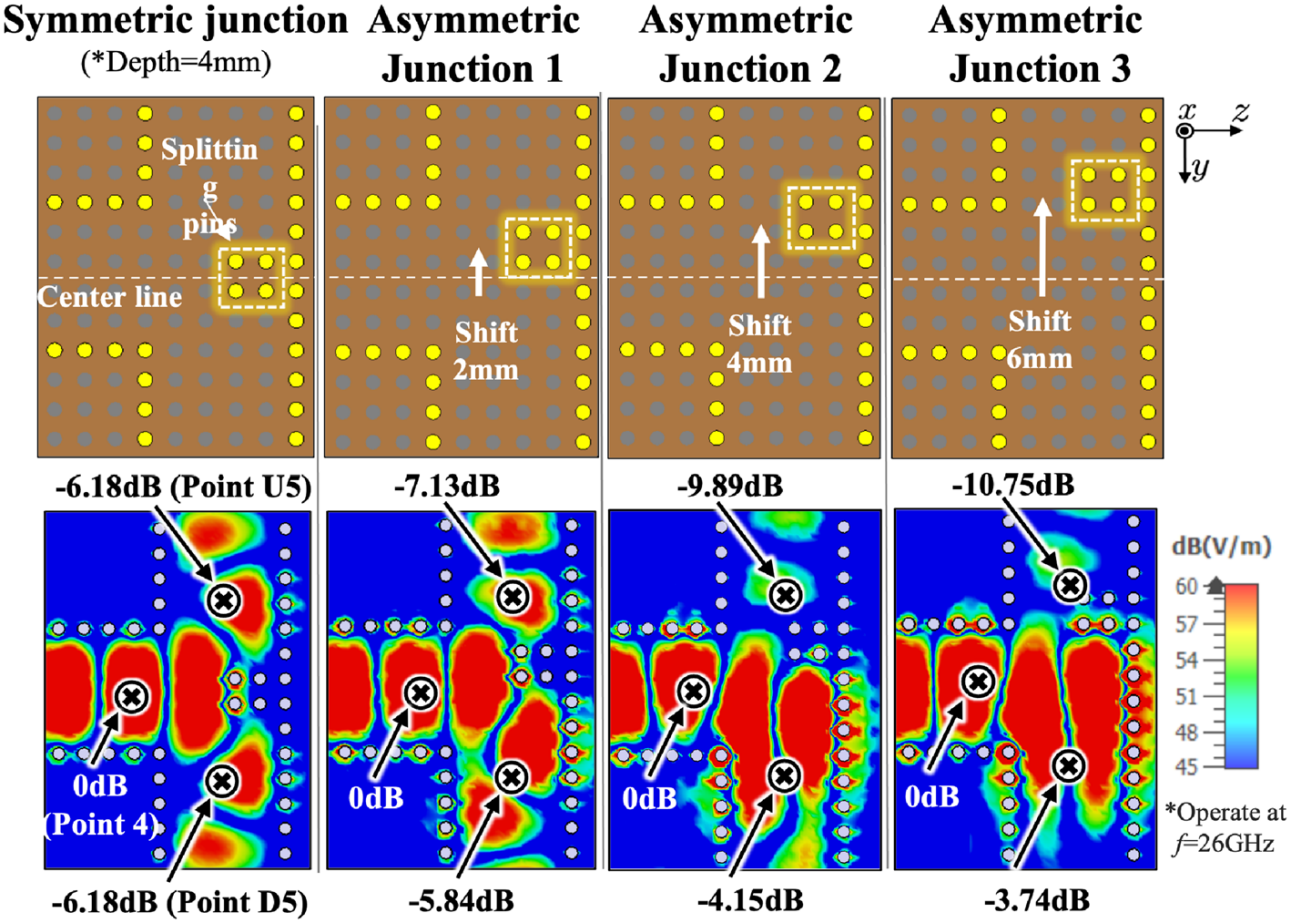
The geometry of symmetric junction and asymmetric junctions 1, 2, 3 with the splitting pins shifted from 2 to 6 mm in the $-y$-direction and their corresponding E-field at Points 4, D5, U5 in three directions of the T-junction at 26 GHz.

### Asymmetric junctions

Figure 9 illustrates the geometries of Asymmetric Junctions 1–3, which are obtained by gradually shifting the splitting pins on the centerline toward the $-y$-direction in a 2 mm step, up to 6 mm, resulting in an asymmetric T-junction. The E-field power at same three sampling points, i.e., Points 4, D5 and U5, are measured. Point 4, with a normalized value of 0 dB, is taken as the reference point as listed in Table 3. In the symmetric junction, the values at D5 and U5 are −6.18 dB, with a ratio of 1:1. In contrast, in Asymmetric Junction 1, the value at Point D5 is −5.84 dB, while that at Point U5 is −7.13 dB, with a ratio of the E-field strengths of 1.3:1. For Asymmetric Junction 2, the difference in the E-field power ratio is enlarged to 4:1, and the ratio further changes to 5.1:1 in Asymmetric Junction 3. These results demonstrate that the power distribution of surface waves at the T-junction of asymmetric geometries varies as the splitting pins are away from the centerline, and more surface waves will be directed towards the wider side of the T-junction. Further moving the pins off the centerline eventually will result in a blockage of the pathway. Overall, these findings illustrate the flexibility of adjusting the position of the splitting pins to control the power distribution of surface waves in different pathways.

### Asymmetric pathways and frequency selectivity

**Figure 10:**
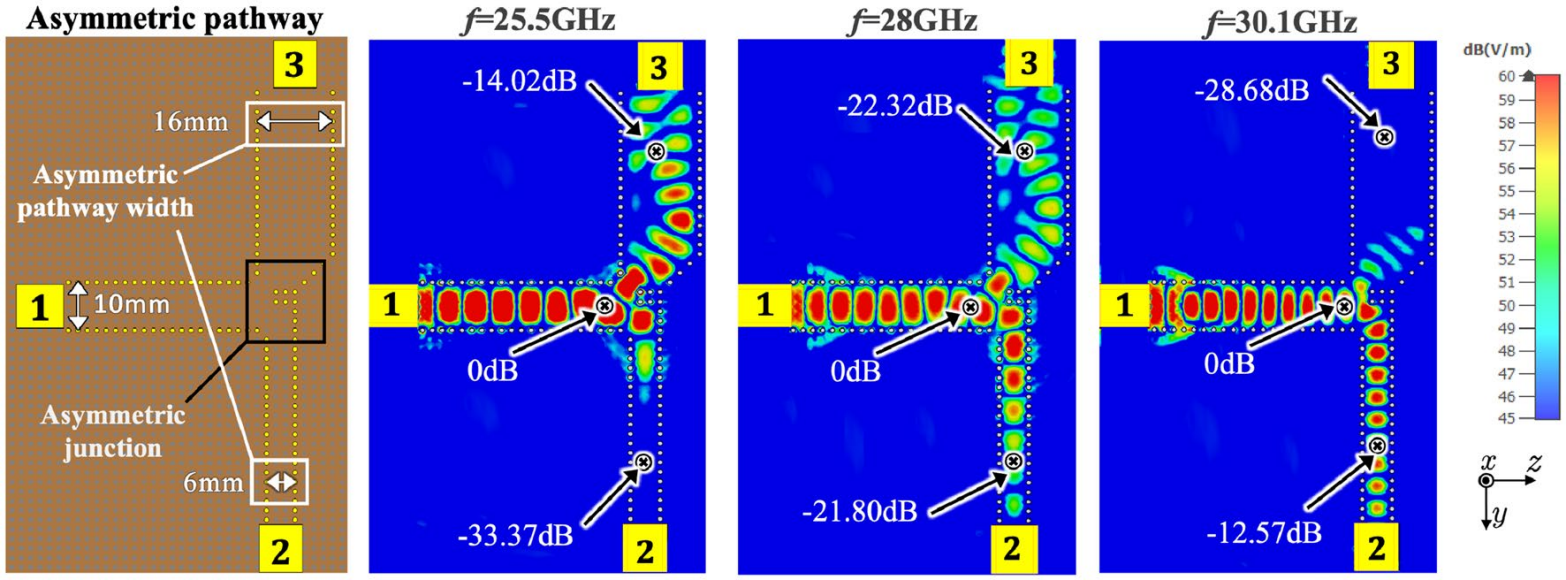
An asymmetric surface geometry with asymmetric widths of 16 mm and 6 mm at upward and downward pathways and its E-field power in simulation results at $25.5\,\textrm{GHz}$, $28\,\textrm{GHz}$ and $-30.1\,\textrm{GHz}$.

**Figure 11:**
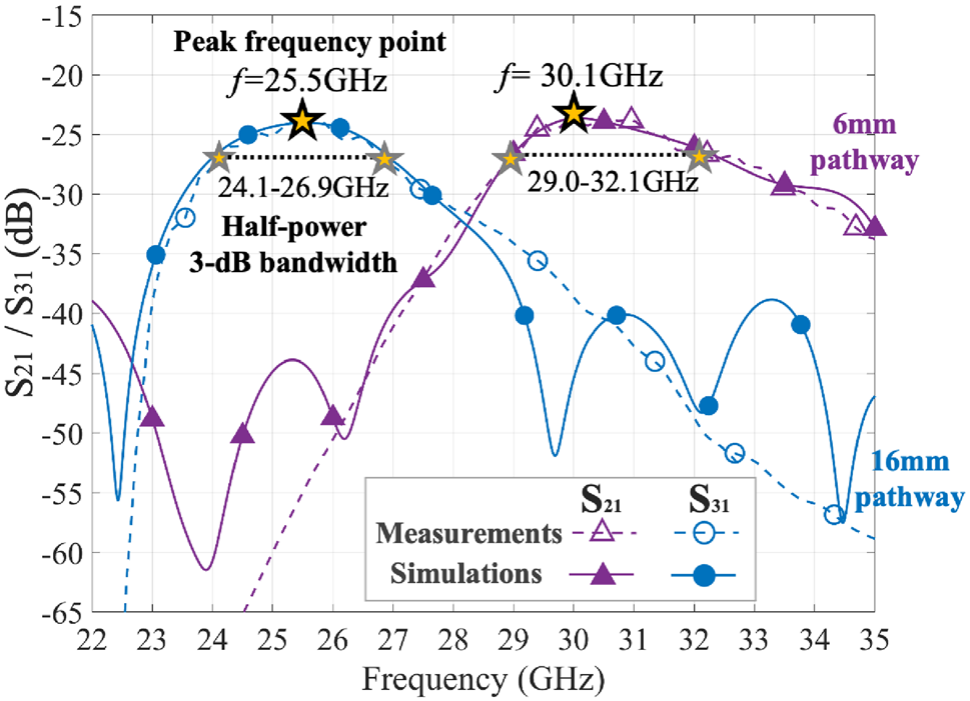
The $\textrm{S}_{21}$/$\textrm{S}_{31}$ (dB) results for an asymmetric surface geometry in the measurements and simulations.

Figure 10 shows an asymmetric pathway example to demonstrate the frequency selectivity of the pathway. The pathway width $w_c$ at the T-junction entrance connecting to Transducer 1 is fixed at 10 mm, while the $+y$- and $-y$-directed pathway widths are set at 16 mm and 6 mm, respectively. The splitting pins in the T-junction are configured asymmetrically to control the power distribution as discussed in “Asymmetric junctions” section. The simulation results indicate that at $f = 25.5$ GHz, $\textrm{S}_{21}$ is $-\,34.17$ dB, while $\textrm{S}_{31}$ is $-\,14.82$ dB, showing an approximately 20 dB difference between Transducers 2 and 3. At 28 GHz, $\textrm{S}_{21}$ is $-\,22.60$ dB, and $\textrm{S}_{31}$ is $-\,23.52$ dB, approaching an equal value approximately. Furthermore, at 30.1 GHz, $\textrm{S}_{21}$ is $-\,13.37$ dB, and $\textrm{S}_{31}$ is −29.68 dB, resulting in a difference of 16 dB between the two ports. These results show that the asymmetric pathway can separate surface waves operating at different frequency bands. The $\textrm{S}_{21}$ and $\textrm{S}_{31}$ of the model in both measurements and simulations are also shown in Fig. 11. The 16 mm wide pathway has an operating frequency of 25.5 GHz and a 3-dB half-power bandwidth from 24.1 to 26.9 GHz. For the 6 mm wide pathway, the operating frequency is 30.1 GHz and the 3-dB half-power bandwidth ranges from 29.0 to 32.1 GHz. At the peak frequency point of each separated pathway, the isolation is about 18 dB. Therefore, the asymmetric pathways have the potential to achieve frequency-based signal distribution by controlling the splitting pins and pathway width simultaneously, allowing surface waves at different frequency bands to be delivered to different transducers. These preliminary results demonstrate the feasibility of flexible control of surface wave in frequency bands. It can be observed that the wider pathway, which is connected to Transducer 3, is not optimized for most efficient surface wave propagation, more comprehensive study is required for more precise frequency division in the next stage.

**Table 3:** The E-field power variations in the symmetric/asymmetric junctions.

Junction	Normalized E-field power (dB)			Difference (dB)	Power ratio
	Point 4	D5	U5	D5-U5	D5:U5
Sym.	0	− 6.18	− 6.18	0	1:1
Asym. 1	0	− 5.84	− 7.13	1.29	1.3:1
Asym. 2	0	− 4.15	− 9.89	5.74	4:1
Asym. 3	0	− 3.74	− 10.75	7.01	5.1:1

## Conclusion

In conclusion, this paper proposes a novel reconfigurable approach for flexibly controlling the power division between surface wave pathways in the millimeter-wave frequency band. The utilization of fluid metal or metal pins at the junction to create splitting pins provides flexibility and adaptability to the system. Through full 3D EM simulation and intensive measurements, this study demonstrates the effectiveness of using splitting pins for distributing surface waves. While varying the apex angles of splitting pin arrangement in the T-junction has little impact on the insertion loss, the distribution of asymmetric splitting pins can aptly distribute the power of surface waves by adjusting the degree of asymmetry. Preliminary results illustrate that adjusting the asymmetric pathway width can separate waves of different frequencies and control the half-power bandwidths of surface waves over different propagation pathways, prompting future research for a more thorough investigation. The results have shown the potential of surface wave technology for possible application in future communication systems.

## Data Availability

The datasets used and/or analysed during the current study available from the corresponding author on reasonable request.
